# A Meta-Analysis of 5-Hydroxytryptamine Receptor 1B Polymorphisms With Risk of Major Depressive Disorder and Suicidal Behavior

**DOI:** 10.3389/fpsyt.2021.696655

**Published:** 2021-07-12

**Authors:** Pingliang Yang, Mengchang Yang, Peng Li, Dejun Cao, Daoyin Gong, Jiahua Lv, Linmei Pu, Sizhou Huang, Yundan Liang

**Affiliations:** ^1^Department of Anesthesiology, The First Affiliated Hospital of Chengdu Medical College, Chengdu, China; ^2^Department of Anesthesiology, Sichuan Academy of Medical Sciences and Sichuan Provincial People's Hospital, Chengdu, China; ^3^Department of Anesthesia, Chengdu Second People's Hospital, Chengdu, China; ^4^Department of Pathology, Hospital of Chengdu University of Traditional Chinese Medicine, Chengdu, China; ^5^Department of Pathology and Pathophysiology, Chengdu Medical College, Chengdu, China; ^6^Development and Regeneration Key Laboratory of Sichuan Province, Department of Anatomy and Histology and Embryology, Chengdu Medical College, Chengdu, China

**Keywords:** HTR1B, polymorphism, major depressive disorder, suicidal behavior, meta-analysis

## Abstract

**Purpose:** Previous association studies have investigated whether genetic polymorphisms in *HTR1B* influenced individuals' susceptibility to major depressive disorder (MDD), anti-depressant response (ADR) and suicidal behavior. However, equivocal evidence was obtained. In this meta-analysis, we aimed to examine the association of *HTR1B* polymorphisms with risk of MDD, ADR and suicidal behavior.

**Materials and Methods:** Studies evaluating the association between *HTR1B* polymorphisms and risk of MDD, ADR and suicidal behavior were searched in Pubmed, Ovid Medline, web of science and China National Knowledge Infrastructure databases. Summary odds ratios (ORs), 95 % confidence intervals (CIs) and *p*-values were calculated using a fixed or random effects model.

**Results:** Meta-analysis findings revealed a significantly increased risk of MDD with rs6296 GC and GC/CC genotypes (GC vs. GG: OR = 1.26, 95% CI, 1.07–1.48; GC/CC vs. GG: OR = 1.22, 95% CI, 1.04–1.43, respectively). Moreover, rs6298 CT genotype was significantly associated with an increased risk of suicidal behavior (CT vs. CC: OR = 1.48, 95% CI, 1.16–1.88). However, both rs6296 and rs130058 were not significant risk factors for lethal suicidal behavior.

**Conclusion:** This meta-analysis identified that rs6296 and rs6298 in *HTR1B* may be significantly related to the risk of MDD and lethality of suicide attempts, respectively. Further studies are required to assess the markers in larger cohorts.

## Introduction

Major depressive disorder (MDD) is a common mental disorder that affects about 216 million people worldwide in 2015 (1). Although a group of anti-depressant agents were used in clinical practice, nearly 30% of patients cannot respond positively to the primary prescription (2, 3) and half of people who died of suicide were related to MDD or other mood disorders (4). The cause of MDD has been demonstrated to be a combination of environmental, psychological and genetic factors (5–8). However, the exact mechanism of genetic contribution in the emergence of MDD and suicidal behavior remains unclear.

The serotoninergic pathway has been implicated to play a crucial role in the pathophysiology of MDD, antidepressant response (ADR) and suicidal behavior (9–12). Some components of this system, such as serotonin receptor 1A, 1B, 2A, 2B, and 2C, are important regulators of metabolism of 5-hydroxytryptamine (5-HT). Among them, 5-HT1B receptor is considered as a nerve terminal autoreceptor, with the function of inhibiting the release of 5-HT. In the hippocampus, activation of postsynaptic 5-HT1B heteroreceptors implicated excitatory synapses as a locus of plasticity in depression (13). The 5-HT1B receptor knockout mice displayed a variety of behavioral paradigms, including lower levels of anxiety (14).

The 5-HT1B receptor is encoded by *HTR1B* gene in humans (15). Previously, genetic association studies have investigated whether single nucleotide polymorphisms (SNPs) in *HTR1B* influenced individuals' susceptibility to MDD and suicidal behavior, such as rs6296, rs6298 and rs130058 (16–27). These SNPs were functional, with rs6296-C allele reducing the level of *HTR1B* mRNA, rs6296G-rs6298C or rs6296 G-rs6298 T haplotype exhibiting higher levels of *HTR1B* mRNA (19), and rs130058 affecting receptor gene activity (28). However, equivocal evidence was obtained. Kao et al. reported that rs6296 GC genotype was significantly associated with an increased risk of MDD and rs6298 CT genotype was significantly associated an increased risk of suicide attempts (19). Conversely, Rujescu et al. reported that there was no significant association of rs6296 with MDD risk (26). The discrepancy may be caused by limited power in a single study with small samples. In order to evaluate the combined evidence from publications, we designed and conducted a meta-analysis examining the association of *HTR1B* polymorphisms with risk of MDD, ADR and suicidal behavior.

## Materials and Methods

### Search Strategy

Followed the PRISMA statement, we screened records by conducting computer-based searches of Pubmed, Ovid Medline (1946–2020), web of science (1900–2020) and China National Knowledge Infrastructure (1915–2020) databases using the following search algorithm: (“5-hydroxytryptamine receptor 1B” or “*HTR1B*”) and (“SNP^*^” or “polymorphism^*^” or “variant^*^” or “susceptibility”) and (“depression” or “major depressive disorder” or “suicide” or “suicidal behavior”). The literature search without language restrictions was carried out up to May 2020.

### Eligibility Criteria

The inclusion criteria for study selection were: (a) association studies reporting *HTR1B* polymorphisms with the risk of MDD or ADR or suicidal behavior and (b) studies published as full-length articles with original genotyping data for computing pooled odds ratios (ORs) and 95% confidence intervals (CIs). The exclusion criteria were: (a) duplicate publications; (b) lack and inaccessibility of data; and (c) meta-analysis. There is no language restriction for inclusion and exclusion criteria.

### Data Extraction

From each included study, the following information was independently extracted by two authors (Pingliang Yang and Mengchang Yang) and checked by a third author (Yundan Liang): name of the first author, year of publication, country, ethnic background of participants, total number and mean age of participants, diagnosis and source of patients, criteria used for matching cases, SNPs studied, genotyping distributions, genotyping method, and quality control for genotyping method. Disagreements were solved by discussion among the three authors.

### Statistical Analysis

A meta-analysis was performed to investigate the association between *HTR1B* polymorphisms and the risk of MDD, ADR and suicidal behavior among different genetic models with available data from at least two independent studies. The distributions of SNP genotypes in controls were retested for conformity to Hardy–Weinberg equilibrium (HWE) using a chi-squared test. The *Q*-test and *I*^2^ metric were used to test heterogeneity across studies (29). In the absence of any detectable between-study heterogeneity (*I*^2^-value <50% and *p* ≥ 0.10), a fixed effects model was used to compute summary ORs and 95% CIs (30); otherwise, a random effects model was used (31). Meta-regression analysis was used to examine potential reasons for between-study heterogeneity. Subgroup analyses were performed to assess the consistency of *HTR1B* polymorphisms among predefined categories of study characteristics such as ethnicity of participants (Asian, Caucasian and mixed population) and outcome of anti-depressant therapy (responder vs. non-response and remitter vs. non-remitter). Publication bias was estimated visually using Egger's linear regression asymmetry test (32). Sensitivity analyses were reported after excluding each study at a time. Data analysis was performed using Stata version 11.0 (Stata Corporation, College Station, TX). Statistical power was calculated using Quanto software version 1.2.3 (Natara Software, Naperville, IL, USA).

## Results

### Flow Diagram of Selection Studies

**Figure 1:**
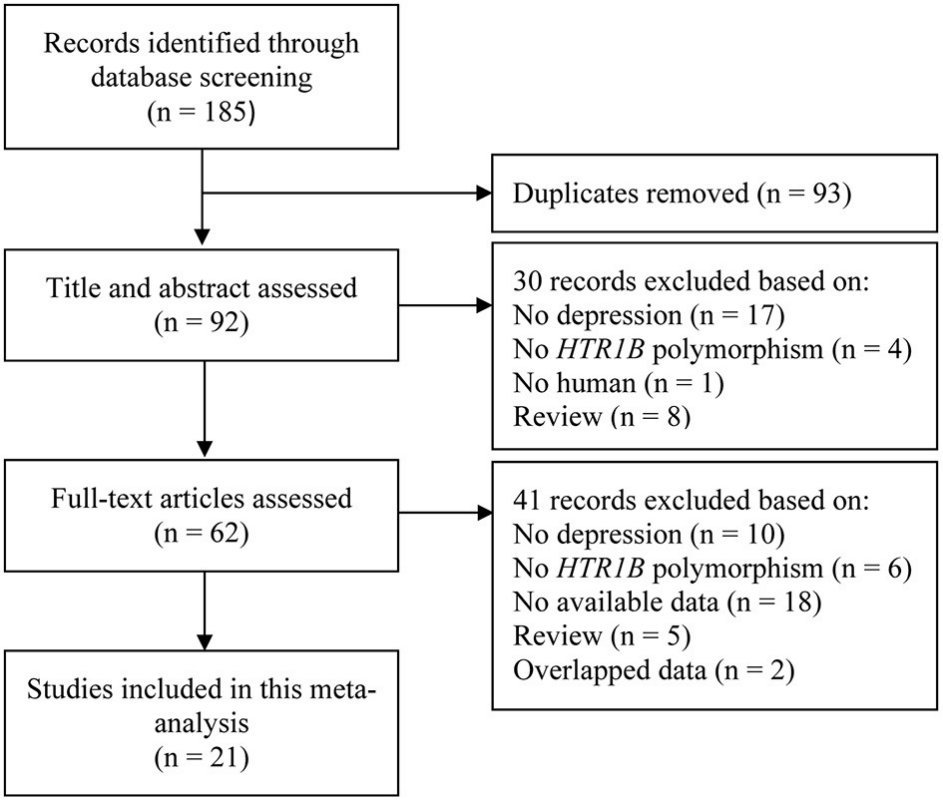
Flow diagram of selection studies.

The flow diagram of selection process is presented in Figure 1. From a total of 185 records retrieved from the primary screening, 92 records were reviewed by title and abstract after removing duplicates (*n* = 93). Thirty records were excluded based on no MDD (*n* = 17), no *HTR1B* polymorphisms (*n* = 4), no human study (*n* = 1) and review articles (*n* = 8). The full-text of the remaining 62 articles was assessed and 41 records were excluded based on no MDD (*n* = 10), no *HTR1B* polymorphisms (*n* = 6), review articles (*n* = 5), no available data (*n* = 18) and duplicate data reporting by the same group (*n* = 2). Finally, 21 studies evaluating the effect of three polymorphisms in *HTR1B* (i.e., rs6296, rs62983 and rs130058) on MDD, ADR and suicidal behavior were included in this meta-analysis.

### Characteristics of Included Studies

The characteristics of the included studies are summarized in Table 1. Of the 21 studies included in this meta-analysis, 6 was conducted in Asians, 11 in Caucasians and 4 in mixed populations reporting more than one ethnic descent. The samples in different studies varied, ranging from 59 to 803. All studies reported genotyping techniques, including polymerase chain reaction (PCR)-restriction fragment length polymorphism, amplification-refractory mutation system, allele-specific PCR, pyrosequencing, Illumina GoldenGate assay and SNaPshot. However, only four studies (19.0%) described matching criteria between cases and controls, and only one study (4.8%) described quality control for genotyping assays.

**Table 1:** Characteristics of literatures included in the meta-analysis.

**References**	**Country**	**Ethnicity**	**Cases**				**Source of controls**	**Matching criteria**	**Genotyping method**	**Quality control**	**Polymorphisms**
			**Number**	**Age**	**Diagnosis and Assessment**	**Source of cases**					
Fehr et al. (16)	Germany	Caucasian	108	-	DSM-IV	MDD patients were recruited from multicenter trails	74 healthy volunteers	-	PCR-RFLP	-	rs6296
Hong et al. (17)	China	Asian	110	37.2 ± 13.3	DSM-IV	Suicidal individuals were defined as those with a history of attempted suicide	215 normal subjects	-	PCR-RFLP	-	rs130058
Huang et al. (18)	USA	Mixed	340	38.9 ± 13.6	DSM-III-R	208 MDD patients and 132 patients with a history of at least one suicide attempt	96 healthy volunteers	Age and sex	PCR-RFLP	-	rs6296
Kao et al. (19)	China	Asian	476	-	DSM-IV	285 MDD patients and 191 MDD patients with a history of suicide attempts	64 were recruited from communities living near the hospital and 249 were individuals with chronic pain	-	ARMS-PCR and sequencing	-	rs6296 and rs6298
Murphy et al. (20)	Ireland	Caucasian	159	34.7	DSM-IV	A suicide attempt was defined as a highly lethal act of self-harm	83 non-attempters	-	Allele specific PCR	-	rs6296
New et al. (21)	USA	Mixed	145	38.± 6 9.8	DSM-IV	40 personality disorder patients with a history of suicide attempts	105 personality disorder patients without a history of suicide attempts	-	PCR-RFLP	-	rs6296
Nishiguchi et al. (22)	Japan	Asian	163	47.9 ± 17.6	-	Suicide victims	163 unrelated volunteers	-	PCR-RFLP	-	rs6296
Noskova et al. (23)	Russia	Caucasian	174	14–72	ICD-10	99 Tatar and 75 Russian patients with unipolar depression	331 volunteers	Age and ethnicity	PCR-RFLP	-	rs6296
Pompili et al. (24)	Italy	Caucasian	111	42.8 ± 12.8	DSM–IV	A suicide attempt was defined as a non-fatal, self-directed, potentially injurious behavior with an intent to die	Non-Suicide Attempter	-	Pyrosequencing	-	rs6296
Pooley et al. (25)	UK	Caucasian	129	38 ± 14 (20–72)	ICD-10	The diagnosis of deliberate self-harm was based on the criteria of the WHO/EURO Multicentre Study of Suicidal Behavior and 78 patients were diagnozed as depressive episode	329 individuals recruited from blood donor clinics	-	Allele specific PCR	Duplicate genotyping	rs6296
Rujescu et al. (26)	Germany	Caucasian	211	39.5 ± 13.3 (18–73)	DSM-IV	148 unrelated suicide attempters and 63 patients with unipolar depressive disorder	327 community-based healthy volunteers	-	PCR-RFLP	-	rs6296
Shaikh et al. (33)	USA	Mixed	201	-	DSM-III or DSM-IV	Adult patients receiving treatment for pertinent mood symptoms during childhood were enrolled	Healthy adults	Sex and ethnicity	PCR-allele specific fluorescent labeled probes	-	rs6296
Silva et al. (34)	Chile	Mixed	59	18–65	DSM-IV	Responders were defined as patients with a reduction of ≥75% in HDRS-17 score after 12 weeks of fluoxetine treatment	-	-	PCR-RFLP	-	rs6296
Słopień et al. (35)	Poland	Caucasian	332	42–67	HRSD	Postmenopausal women with depressive disorder	219 postmenopausal women	-	PCR-RFLP	-	rs6296
Stefulj et al. (36)	Croatia	Caucasian	363	49 ± 19	-	Suicide completers	440 individuals without personal or family history of neuropsychiatric disorders	-	PCR-RFLP	-	rs6296
Tsai et al. (37)	China	Asian	160	43.9 ± 16.4 (18–74)	DSM-IV	MDD patients received a 4-week fluoxetine treatment and response was defined as a minimum reduction of 50% in HAMD score	160 normal subjects		PCR-RFLP	-	rs130058
Videtic et al. (38)	Slovenia	Caucasian	226	49.6 ± 17.3	-	Suicide victims	225 blood donors with no history of neuropsychiatric disorders	-	PCR-RFLP	-	rs6296 and rs130058
Wang et al. (39)	China	Asian	85	36.7 ± 14.1	HAMD-17	Patients were treated with single antidepressant escitalopram and 47.1% of the patients achieved remission. Clinical remission was defined as patients with a HAMD score ≤ 7 or reduction of ≥75% in HAMD score after 8 weeks of treatment.	-	-	PCR-RFLP and PCR-sequencing	-	rs6296, rs6298, rs1228814 and rs1778258
Wilkie et al. (40)	UK	Caucasian	268	43.4 ± 11.3 (18–65)	ICD-10 and DSM-IV	166 patients with unipolar depression and 102 patients with early onset depression	-	-	PCR-RFLP	-	rs6296 and rs130058
Xu et al. (41)	China	Asian	308	18–60	DSM-IV and HDRS-17	Responders were defined as patients with a reduction of ≥50% in HDRS-17 score after 12 weeks of treatment	-	-	Illumina GoldenGate assay	-	rs6298
Zouk et al. (42)	Canada	Caucasian	338	38 ± 11	-	Suicide completers	358 controls without a history of suicidal behavior or major psychiatric diagnoses	-	SNaPshot	-	rs6296, rs6298, rs130058,−261T/G and 1180A/G

*DSM, diagnostic and statistical manual of mental disorders; MDD, major depressive disorder; PCR-RFLP, polymerase chain reaction-restriction fragment length polymorphism; ARMS, amplification-refractory mutation system; ICD, international classification of diseases; WHO/EURO, world health organization/European; HRSD, Hamilton rating scale for depression; HAMD, Hamilton depression rating scale*.

Eight studies investigated the association between *HTR1B* rs6296 polymorphism and MDD risk, involving 1,230 cases and 1,863 controls, with a statistical power of 93.5% when setting the relative ratio as 1.3 under a dominant model. The rs6296 genotype distributions in controls of all studies conformed to HWE. The frequency of rs6296 C allele was 43.7% in Chinese population, 27.9% in Caucasians and 24.0% in mixed population. Two studies investigated the effect of *HTR1B* rs6296 polymorphism on the outcome following antidepressant therapy. Regarding the relationship between *HTR1B* polymorphisms and the risk of suicidal behavior, 11 studies focused on SNP locus rs6296, 3 studies focused on rs130058 and 2 studies focused on rs6298, with statistical power of 99.9, 83.5, and 80.8%, respectively.

### Meta-Analysis

**Figure 2:**
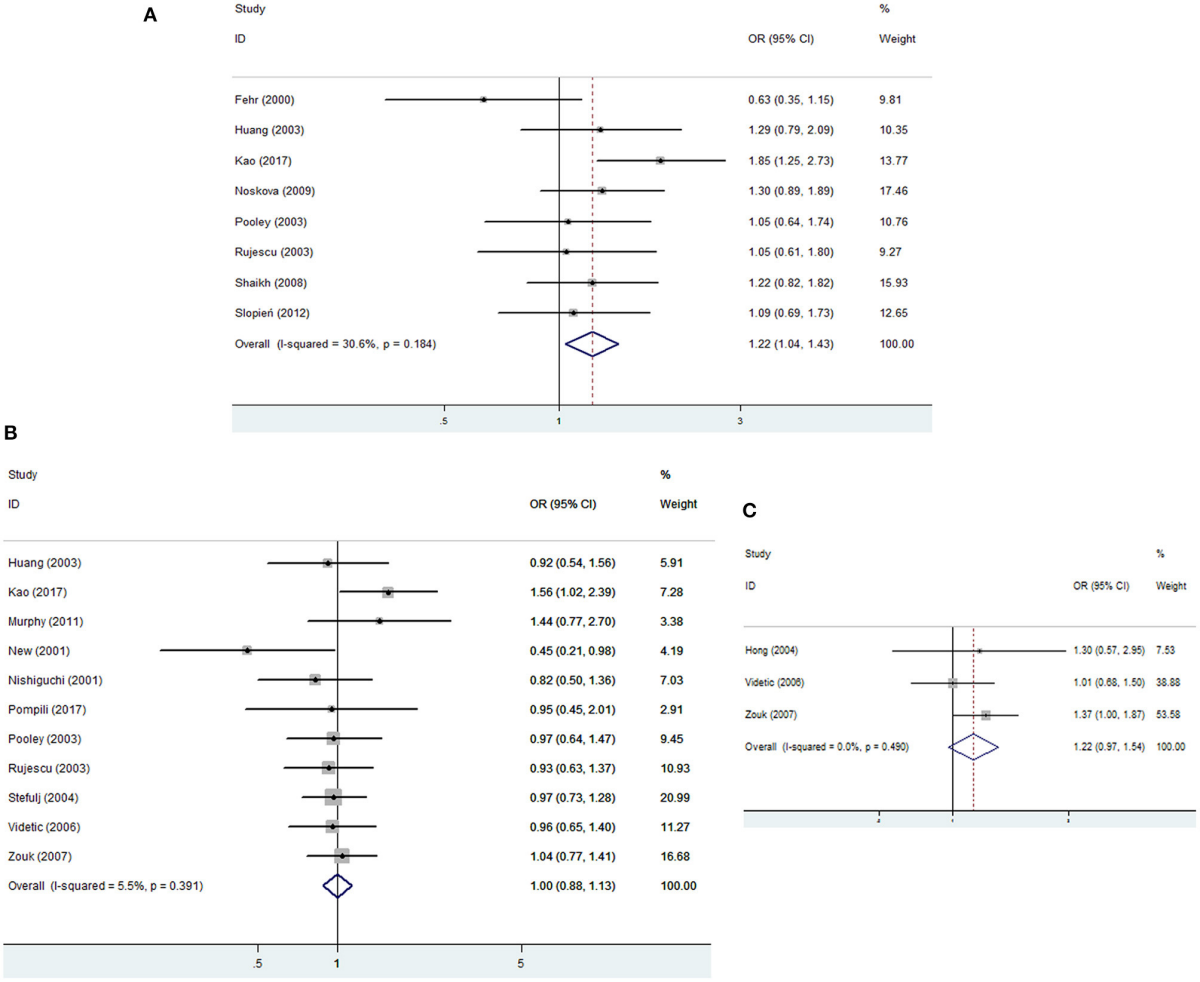
Meta-analysis of *HTR1B* polymorphisms with the risk of MDD and suicidal behavior. **(A)** rs6296 polymorphism and MDD risk; **(B)** rs6296 polymorphism and the risk of suicidal behavior; **(C)** rs130058 polymorphism and the risk of suicidal behavior. A fixed effects model was used under a dominant model.

Pooled ORs for the genotypic and allelic comparisons and corresponding *p*-value for heterogeneity test are presented in Tables 2, 3. In overall analysis of the association between rs6296 and MDD risk, no obvious heterogeneity was detected, and thus a fixed effects model was used. Meta-analysis findings revealed a significantly increased risk of MDD with rs6296 GC and GC/CC genotypes (GC vs. GG: OR = 1.26, 95% CI, 1.07–1.48; GC/CC vs. GG: OR = 1.22, 95% CI, 1.04–1.43, respectively) (Figure 2A). Subgroup analysis of studies by ethnicity (Caucasians and mixed population) did not reveal any significant association between rs6296 and MDD risk. Following anti-depressant treatment, the difference of the rs6296 genotype frequencies was observed neither in remitters and non-remitters nor in responders and non-responders (Table 2).

**Table 2:** Meta-analysis of *HTR1B* rs6296 polymorphism and the risk of MDD and ADR.

	**n ^**a**^**	**Heterozygous comparison**		**Homozygous comparison**		**Dominant model**		**Recessive model**		**Allele comparison**	
		**OR (95% CI)**	***P_**−**_*value^**b**^**	**OR (95% CI)**	***P_**−**_*value^**b**^**	**OR (95% CI)**	***P_**−**_*value^**b**^**	**OR (95% CI)**	***P_**−**_*value^**b**^**	**OR (95% CI)**	***P_**−**_*value^**b**^**
**MDD vs. control**											
Total	8	1.26 (1.07–1.48)	0.18	1.07 (0.80–1.45)	0.26	1.22 (1.04–1.43)	0.18	0.87 (0.66–1.14)	0.19	1.09 (0.97–1.23)	0.38
**Ethnicity**											
Caucasian	5	1.09 (0.87–1.36)	0.67	0.96 (0.63–1.45)	0.10	1.06 (0.86–1.31)	0.40	0.92 (0.62–1.38)	0.16	1.03 (0.87–1.21)	0.17
Mixed	2	1.23 (0.89–1.69)	0.70	1.38 (0.70–2.75)	0.47	1.25 (0.92–1.70)	0.87	1.27 (0.65–2.49)	0.41	1.19 (0.93–1.53)	0.85
**Responder vs. non-responder**											
Total	2	0.71 (0.47–1.07)	0.37	1.59 (0.74–3.40)	0.89	0.81 (0.55–1.19)	0.44	1.75 (0.84–3.63)	0.64	0.97 (0.71–1.32)	0.59
**Remitter vs. non-remitter**											
Total	2	0.73 (0.47–1.12)	0.91	0.94 (0.46–1.95)	0.91	0.77 (0.51–1.15)	0.96	1.13 (0.59–2.15)	0.91	0.88 (0.65–1.20)	0.73

*MDD, major depressive disorder; ADR, antidepressant response; OR, odd ratio; CI, confidence interval*.

a *number of studies;*

b *Q-test of heterogeneity*.

**Table 3:** Meta-analysis of *HTR1B* polymorphisms and the risk of suicide behavior.

**Polymorphisms**	**n ^**a**^**	**Heterozygous comparison**		**Homozygous comparison**		**Dominant model**		**Recessive model**		**Allele comparison**	
		**OR (95% CI)**	***P_**−**_*value^**b**^**	**OR (95% CI)**	***P_**−**_*value^**b**^**	**OR (95% CI)**	***P_**−**_*value^**b**^**	**OR (95% CI)**	***P_**−**_*value^**b**^**	**OR (95% CI)**	***P_**−**_*value^**b**^**
**rs6296**											
Total	11	1.02 (0.86–1.22)^c^	0.09	0.89 (0.70–1.12)	0.97	1.00 (0.88–1.13)	0.39	0.88 (0.71–1.10)	0.60	0.98 (0.88–1.07)	0.96
**Ethnicity**											
Caucasian	7	1.03 (0.90–1.17)	0.70	0.83 (0.61–1.23)	0.86	1.00 (0.86–1.15)	0.95	0.82 (0.61–1.10)	0.70	0.97 (0.86–1.09)	1.00
Asian	2	1.13 (0.47–2.71)^c^	0.01	0.81 (0.36–1.84)	0.77	1.15 (0.61–2.15)^c^	0.06	0.96 (0.46–2.00)^c^	0.05	1.06 (0.87–1.29)	0.94
Mixed	2	0.70 (0.45–1.11)	0.11	1.04 (0.67–1.60)	0.72	0.73 (0.47–1.12)	0.14	0.96 (0.43–2.16)	0.98	0.81 (0.57–1.14)	0.24
**rs130058**											
Total	3	1.28 (1.00–1.63)	0.45	1.02 (0.69–1.50)	0.83	1.22 (0.97–1.54)	0.24	0.92 (0.64–1.31)	0.90	1.09 (0.92–1.29)	0.55
Caucasian	2	1.27 (0.98–1.65)	0.21	1.01 (0.68–1.49)	0.66	1.22 (0.95–1.55	0.49	0.91 (0.63–1.31)	0.94	1.08 (0.91–1.29)	0.30
**rs6298**											
Total	2	1.48 (1.16–1.88)	0.14	1.31 (0.82–2.09)	0.12	1.49 (0.98–2.25)^c^	0.08	1.09 (0.69–1.71)	0.25	1.29 (0.95–1.75)^c^	0.09

*MDD, major depressive disorder; OR, odd ratio; CI, confidence interval*.

a *number of studies;*

b *Q-test of heterogeneity;*

c *Pooled ORs were computed using the Random-effects model*.

For *HTR1B* polymorphisms with the risk of suicidal behavior, meta-analysis findings revealed that rs6298 CT genotype was significantly associated with an increased risk of suicidal behavior (CT vs. CC: OR = 1.48, 95% CI, 1.16–1.88). However, both rs6296 and rs130058 were not significant risk factors for lethal suicidal behavior in overall and subgroup analysis in terms of ethnicity (Table 3, Figures 2B,C).

### Evaluation of Heterogeneity

In the heterozygous comparison of rs6296 with the risk of suicidal behavior, obvious heterogeneity was observed, and then meta-regression analysis was used to examine the source of heterogeneity by ethnicity (Asian, Caucasians and mixed population), genotyping methods and sample size (>200 and ≤ 200). However, none of these variables can explain the heterogeneity, indicating that there are some unknown factors influencing the heterogeneity.

### Sensitivity Analysis

The effect of a single study on summary ORs was evaluated by exclusion of one study each time and statistically similar results were obtained, indicating stability of the pooled ORs.

### Publication Bias

**Figure 3:**
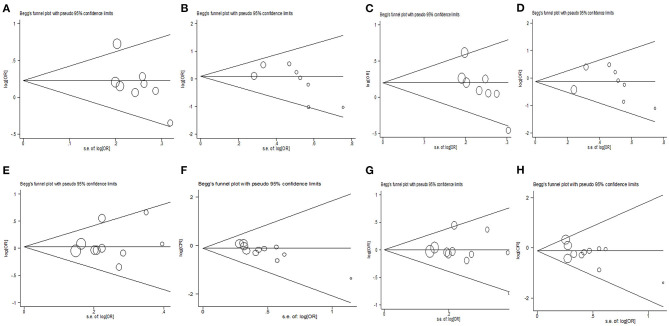
Egger's funnel plot for publication bias test of *HTR1B* rs6296 polymorphism with MDD **(A–D)** and suicidal behavior **(E–H)**. **(A,E)** GC vs. GG; **(B,F)** CC vs. GG; **(C,G)** GC/CC vs. GG; **(D,H)** CC vs. GG/GC.

Egger's linear regression asymmetry test was used to estimate the publication bias. For publications investigating the association between rs6296 and MDD risk, the funnel plot showed a significance in a dominant genetic model (*p* = 0.02). The bias disappeared after excluding the study with sample size <200 (16) and the overall effect of rs6296 on MDD risk remains unchanged (OR = 1.28, 95% CI: 1.09–1.51). For publications investigating the association between rs6296 and the risk of suicidal behavior, the funnel plot also showed a significance in homozygous comparison (*p* = 0.001). The bias disappeared when excluding the studies with sample size <200 (20, 21, 24) and the overall effect of rs6296 on the risk of suicidal behavior remains unaltered (OR = 0.94, 95% CI: 0.73–1.21) (Figure 3).

## Discussion

5-HT1B receptor is a nerve terminal autoreceptor that is involved in the modulation of 5-HT synthesis and release in rat brain cortex (43). Mice absence of 5-HT1B receptor were observed to exhibit altered psychiatric disorders, such as increased aggression, alcohol and cocaine intake, as well as lower levels of anxiety (14, 44–46). Growing evidence has shown that 5-HT1B receptor is implicated in the pathophysiology of MDD, serving as a promising new target for antidepressant treatment (47, 48). *HTR1B*, encoding 5-HT1B receptor, is located on 6q14.1 in human genome. Three common genetic polymorphisms in *HTR1B* (i.e., rs6296, rs6298, and rs130058) have been widely examined in affective disorders. However, results from previous individual studies are contradictory regarding the role of *HTR1B* polymorphisms in MDD and suicidal behavior. A meta-analysis is therefore valuable with the objective of offering a comprehensive assessment of the effect of *HTR1B* polymorphisms on the etiology of MDD and suicidal behavior. Our meta-analysis showed that carriers with rs6296 GC and GC/CC genotypes had a 1.26- and 1.22-fold increased risk of MDD, respectively, and carriers with rs6298 CT genotype had a 1.48-fold increased risk of suicidal behavior. Our study has more than 80% power statistics, indicating the results were solid. These findings suggested that both rs6296 and rs6298 in *HTR1B* were significant genetic risk factors for the development and progression of MDD, which was consistent with the data from genome-wide association study (49).

As for rs6296 in *HTR1B*, no previous meta-analysis of the genetic variant in the onset of MDD has been published to date. This meta-analysis including 8 studies (1,230 cases and 1,863 controls) showed a significantly increased risk of MDD (16, 18, 19, 23, 25, 26, 33, 35), supporting previous results reported by Kao et al. who found that age and rs6296 GC genotype were significantly associated with MDD (19). A possible mechanism of rs6296 enhancing MDD risk is that patients with the rs6296-C allele had lower levels of *HTR1B* mRNA, displayed more hostility and aggressive behavior, and finally caused a higher risk of MDD and suicidal ideation (19). According to data from 1,000 Genomes, the distribution of rs6296 C allele varied largely among different ethnicities, with a frequency of 50.9% in East Asian, 26.3% in European and 24.4% in African. The distribution variance of rs6296 in different ethnic groups may influence the risk of MDD. We therefore performed subgroup analysis based on ethnicity (Asian, Caucasian and mixed population). However, we did not identify positive effect of rs6296 on MDD risk among Asian, Caucasian and mixed population. These results should be interpreted cautiously because only two studies were performed in mixed population (18, 33) and only one study was performed in Asian (19). To make the results robust, further evidence for a relationship between rs6296 and MDD in diverse ethnic groups is required.

Currently, antidepressants are the first line of treatment for MDD. Conflicting results were obtained regarding the effectiveness of antidepressants (50). Previous work has attempted to find out whether rs6296 in *HTR1B* was a biological predictor of antidepressant response (34, 39, 40). The statistical power in each study was insufficient due to small sample sizes, which may lead to type II error and false negative findings. For example, only 49 patients including 22 responders and 27 non-responders were genotyped in Amerindians and Caucasians (34). In this meta-analysis, we reevaluated the effect of rs6296 on the response of antidepressant medication by pooling all the published data together, and we failed to find any significant association of the rs6296 with antidepressant treatment. Even though we added up all the available samples, the total number is still very small and the power may not be strong enough yet for statistical significance. Future studies are necessary to validate the role of *HTR1B* polymorphisms in pharmacological response to antidepressants.

Globally, MDD is the major reason for suicide, with about 50% of people dying of suicide having MDD or other mood disorders (4, 51). And thus it is of value to examine whether the MDD-risk factor rs6296 is linked to suicidal behavior. After records screening and eligibility process, 11 articles were included in this meta-analysis, involving 1,844 cases and 2,479 controls. Our meta-analysis revealed that rs6296 was not a significant risk factor for lethal suicidal behavior in overall analysis as well as subgroup analysis in terms of ethnicity. These findings were in accordance to a previous meta-analysis conducted in 2007 (52). We cannot rule out the possibility of selection bias of participants that caused the negative results. For instance, the controls in Murphy's study deviated from HWE (20).

Besides rs6296, two other SNPs in *HTR1B* (i.e., rs130058 and rs6298) were also investigated in suicide completers. Then meta-analysis was performed, and we found that the rs6298 rather than rs130058 was significantly associated with an increased risk of suicidal behavior in a heterozygous comparison. Previous work has revealed that rs6296 and rs6298 displayed linkage disequilibrium (19), and thus it is difficult to understand how these SNPs might have different clinical consequences. We have to admit that the results may occur by chance due to limited studies included in this meta-analysis. Therefore, the relationship of rs6296, rs6298, and rs130058 with suicidal behavior will be an important issue that needs to be addressed in further studies.

Heterogeneity among studies was observed in the heterozygous comparison of rs6296 with the risk of suicidal behavior. Some common candidates such as ethnicity, genotyping methods and sample size (>200 and ≤ 200) were not demonstrated to affect heterogeneity, indicating that uncontrolled confounding factors and inherent selection bias may explain the heterogeneity. Publication bias observed in a few comparisons may be attributed to small sample size since the bias disappeared when excluding the studies with sample size <200. The results remained unaltered after omission of these studies, suggesting that the true association was not overestimated by selective publication of positive results.

Notably, most of the published studies focused on the effect of rs6296 on the risk of MDD and suicidal behavior. Only two studies investigated whether rs6296 influenced the outcome of anti-depressant treatment and whether rs6298 influenced the risk of suicidal ideation. Moreover, very limited studies included in our meta-analysis reported detailed information, such as matching criteria between cases and controls and quality control for genotyping assays. Given these limitations in this meta-analysis, genetic association studies are be of great importance using well-designed multi-center cohorts with large samples.

In summary, this meta-analysis identified that rs6296 in *HTR1B* was significantly associated with the risk of MDD and rs6298 in *HTR1B* was significantly associated with the risk of suicidal behavior. Further studies investigating the relative contribution of SNPs in *HTR1B* and the mechanisms by which they affect the susceptibility of developing MDD, ADR and suicidal behavior are warranted.

## Data Availability Statement

The original contributions presented in the study are included in the article/supplementary material, further inquiries can be directed to the corresponding author/s.

## Author Contributions

YL and SH designed the study and wrote the manuscript. PY and MY managed the literature searches and analyses. PL, DC, DG, LP, and JL undertook the statistical analysis. All authors contributed to and have approved the final manuscript.

## Conflict of Interest

The authors declare that the research was conducted in the absence of any commercial or financial relationships that could be construed as a potential conflict of interest.
